# Working Memory as an Indicator for Comparative Cognition – Detecting Qualitative and Quantitative Differences

**DOI:** 10.3389/fpsyg.2020.01954

**Published:** 2020-08-06

**Authors:** Lukas Alexander Hahn, Jonas Rose

**Affiliations:** Neural Basis of Learning, Institute of Cognitive Neuroscience, Faculty of Psychology, Ruhr University Bochum, Bochum, Germany

**Keywords:** Macphail, null-hypothesis, quantifying cognition, models of working memory, comparative cognition

## Abstract

Working memory (WM), the representation of information held accessible for manipulation over time, is an essential component of all higher cognitive abilities. It allows for complex behaviors that go beyond simple stimulus-response associations and inflexible behavioral patterns. WM capacity determines how many different pieces of information (items) can be used for these cognitive processes, and in humans, it correlates with fluid intelligence. As such, WM might be a useful tool for comparison of cognition across species. WM can be tested using comparatively simple behavioral protocols, based on operant conditioning, in a multitude of different species. Species-specific contextual variables that influence an animal’s performance on a non-cognitive level are controlled by adapting the WM paradigm. The neuronal mechanisms by which WM emerges in the brain, as sustained neuronal activity, are comparable between the different species studied (mammals and birds), as are the areas of the brain in which WM activity can be measured. Thus WM is comparable between vastly different species within their respective niches, accounting for specific contextual variables and unique adaptations. By approaching the question of “general cognitive abilities” or “intelligence” within the animal kingdom from the perspective of WM, the complexity of the core question at hand is reduced to a fundamental memory system required to allow for complex cognitive abilities. This article argues that measuring WM can be a suitable addition to the toolkit of comparative cognition. By measuring WM on a behavioral level and going beyond behavior to the underlying physiological processes, qualitative and quantitative differences in cognition between different animal species can be identified, free of contextual restraints.

## Introduction

In his article on vertebrate intelligence, Macphail (1985) argues that there are no qualitative differences between vertebrate species when it comes to their cognitive abilities. His major line of reasoning builds on the success of the “Columban Simulation Project” to reproduce experiments performed with primates, using pigeons (Epstein, 1981, 1986; Epstein et al., 1984). While quantitative differences can be described, e.g., by inspecting the required amount of training to master a task (Scarf and Colombo, 2020), the claim for a lack of qualitative difference appears to be more robust. Macphail identifies contextual variables [species-specific experimental conditions, a notion also investigated by Bitterman (1975)] as the source of perceived qualitative differences amongst vertebrates. Neutralizing them would reveal cognitive abilities to be identical.

A prominent approach for comparative cognition features experiments that have shown the impressive abilities of higher cognition of primates, and apply these experiments to other species. Amongst those are protocols that train the animal to apply an abstract rule to a novel set of stimuli. This is the case for numerical competence (Brannon and Terrace, 1998) and orthographic processing (Grainger et al., 2012), to name but two examples. Other experiments focus on an innate cognitive ability that should be present without training a specific response. Famous examples are the mirror self-recognition test (Gallup, 1970) and experiments testing “theory of mind” (Hare et al., 2001). While it is not impossible to train animals other than primates on these tasks (Reiss and Marino, 2001; Plotnik et al., 2006; Clayton et al., 2007; Prior et al., 2008; Scarf et al., 2011, 2016), the experiments might contain insurmountable methodological hurdles for some vertebrate species (e.g., the task may require more training than is possible in animals with short life-span or hands to manipulate objects or operate a touch-screen). These hurdles may prevent an animal from performing successfully in the task, sometimes leading to ambivalent results (Call and Tomasello, 2008; de Waal, 2019). Bitterman focused on qualitative differences of cognition regarding learning (Bitterman, 1975). For example, monkeys and rats maximized reward in probability learning, pigeons and fish showed probability matching (Bitterman et al., 1958; Bullock and Bitterman, 1962b; Wilson et al., 1964; Woodard and Bitterman, 1973). For habit reversal pigeons and rats showed progressive improvement while fish did so only under specific circumstances (Bitterman et al., 1958; Bullock and Bitterman, 1962a; Engelhardt et al., 1973). Together these tests represent a large family (also including tests of inhibitory control, object permanence, and social cognition) that are a set of tools with which complex cognitive behavior can be described and its intricacies can be investigated and disentangled. We suggest adding a different approach to this family. One that investigates a fundamental trait of cognition on a physiological level that can be compared between species. To that end, we suggest working memory (WM) as a critical component of many higher cognitive functions. This addition is warranted by findings of comparative behavioral science and neuroscience. Despite their long independent evolution (Hedges, 2002) resulting in vastly different brain architectures, the cognitive abilities of mammals and birds are very similar, a case of convergent evolution (Colombo and Scarf, 2012; Güntürkün, 2012; Güntürkün and Bugnyar, 2016; Nieder, 2017). WM has been investigated on a behavioral and a physiological level in birds and compared to mammalian WM (Veit and Nieder, 2013; Ditz and Nieder, 2015; Balakhonov and Rose, 2017; Rinnert et al., 2019; Fongaro and Rose, 2020). The bottom line under all these investigations is that besides the different organizations of their brains the same fundamental processes take place. A comparison of different species at this physiological level of WM would widen the scope of comparative cognition and would allow testing Macphail’s idea that focusses primarily on learning, in a new way. Differences and similarities in WM (e.g., in its capacity) may offer insights into why some animals may be (un-)able to display certain cognitive behaviors. Macphail’s null-hypothesis can thus be investigated in the light of potentially qualitative, and quantitative differences of a fundamental trait of cognition.

Using WM comes with its own set of challenges: a precise definition, its concrete link with higher cognition, a precise measurement of the process, and control of the testing environment are all required to ensure a comparable metric. If those challenges are met, WM can be a powerful tool to determine quantitative or even qualitative differences in cognition amongst vertebrates.

**BOX 1. Different approaches of measuring WM.**

There are two different approaches for measuring WM in behavioral experiments, focusing on different aspects of the concept. In the first approach, WM is operationalized as both temporary storage of information (in a range from seconds to hours) and executive functions for retrieval and manipulation of this information (Baddeley, 1992; Floresco and Phillips, 2001; Phillips et al., 2004). This means WM contains accessible information, up to hours after the information has been initially available, and even if the animal, at acquisition, did not know it would require the information later on. The approach is commonly tested in rodents (most prominently rats) and on occasion in other species (e.g., fish and pigeons). Animals are usually tested in a spatial context and over retention intervals ranging from a few seconds to hours (e.g., Olton and Samuelson, 1976; Roitblat et al., 1982; Roberts and Van Veldhuizen, 1985). The tasks the animals perform often consist of navigation in an open area or a radial arm maze in search of food. Efficient navigation of the maze requires memorizing which parts have already been visited (and hence are depleted of food) and visiting only those that have not yet been visited still containing food.

In the second approach, WM is measured as a form of actively sustained short-term memory. In this case, the animal knows that the information will be behaviorally relevant soon and has to hold it accessible and subject it to manipulation, during and after a short delay, in the range of a few seconds (Goldman-Rakic, 1995). Monkeys and birds (but also rodents) are usually tested in tasks during which actively sustained WM can be measured, requiring the animal to attend to a stimulus, remember it and perform a discriminative choice based on the retained stimulus after a short delay of a few seconds (e.g., monkeys: (Fuster and Alexander, 1971), pigeons: (Diekamp et al., 2002), crows: (Veit et al., 2014), mice: (Wu et al., 2020), rats: (Baeg et al., 2003; Tsutsui et al., 2016). The active memory component in these tasks bridges the temporal gap in which the stimulus is not present and holds the information accessible. The absence of the stimuli defines the duration of WM.

These approaches can be challenging to disentangle at the behavioral level since they build on the same definition of working memory introduced by Baddeley and Hitch (1974). However, it is important to keep in mind that these approaches can imply different neural mechanisms. Active maintenance was never demonstrated, and seems counterintuitive, for maintenance that lasts several hours, but the term WM has been used for such long delays. Likewise, many animal studies of WM utilizing a delayed matching to sample task do not directly demonstrate an executive component, which makes it difficult to distinguish behaviorally between short term and working memory. We favor a definition of WM as the cognitive effort of actively keeping stimulus information in an accessible state that can be manipulated for cognitive processes while the stimulus is not physically present. This definition implies a testable neural fingerprint (sustained physiological activity), short duration, susceptibility to interference (Box 2), and limited capacity. These are all aspects that allow for a quantification of WM in different species while the definitive neural fingerprint, active maintenance, can provide a qualitative test if *this WM* is present in a given species.

**BOX 2. Influences on WM.**

Proactive and retroactive interference:

When testing WM, both proactive (e.g., Grant, 1975) and retroactive interference can occur. Our definition makes the measuring of WM robust to such interference. The physiological trace is informative about which stimulus sample is encoded, thus stimuli from both sources of interference can be differentiated in terms of cause for memory failure.

Encoding of preceding sample stimuli is reflected in the activity of individual neurons that show specific sustained activity during the delay that corresponds to correct and incorrect choice behavior. For example, neuron N has elevated sustained activity during the delay following the presentation of sample A and baseline activity following the presentation of sample B. Another neuron AN shows sustained activity following B but only baseline activity following A. On correct trials with sample A, N has sustained activity, AN does not, and the animal matches the sample. On error trials, with sample A, N has baseline activity and AN has sustained activity, subsequently the animal mismatches the sample by choosing B. This has been shown in both monkeys and crows (Brody et al., 2003; Nieder, 2012; Veit et al., 2014; Moll and Nieder, 2015). The same holds on a more abstract level for neurons encoding different behavioral rules like “match” or “non-match,” instead of purely sensory stimuli (Wallis et al., 2001; Veit and Nieder, 2013). In this way, if e.g., trial one was correct with sample A, trial two was correct with sample B, and trial three was incorrect with sample A, the possible interference of sample B with WM of sample A can be detected. This also fits the conclusion of Grant about prospective interference that “the retention deficit in pigeon STM is the product of competition between the prior, conflicting memory and the current memory at the time of the Trial 2 test” (Grant, 1975, p. 214).

The effect of retroactive interference on WM during the delay (in the form of distractors) has been detected in the physiological trace of neurons. During the presentation of the distractor, information about the sample (i.e., differential neuronal activity) diminishes, and the distractor is encoded in neuronal activity (Miller et al., 1996; Jacob and Nieder, 2014). In the following delay, sample information recovers, while distractor information is not sustained (Miller et al., 1996; Jacob and Nieder, 2014). Memory failure (mismatching the sample) correlates with the decay of sample information following the distractor presentation, while the information about the distractor does not have any influence (Jacob and Nieder, 2014). Additionally, if instead of a distractor the sample is repeated, information about the sample increases (Jacob and Nieder, 2014).

*Differential behavior during the delay*:

Differential behavior (e.g., Zentall et al., 1978) can be used as a strategy to avoid the use of WM as we define it. Its presence should thus result in the absence of the WM trace (sustained activity), while behavioral performance solves the task. Subsequently, if there is no WM trace during the delay of the task, the experimental parameters must be adjusted to prohibit alternative behavior-mediated strategies.

## A Definition of Working Memory

The concept of WM was originally devised by Baddeley and Hitch (1974), developed from the earlier models of short term memory of Broadbent (1958) and Atkinson and Shiffrin (1968). Fundamentally, WM is the process of holding limited information accessible for a limited time. Importantly, this maintenance is controlled by executive processes that also allow for the manipulation of this information (Honig, 1978; Baddeley, 2000; Cowan, 2014). A common test in humans is the n-back task (Conway et al., 2005), here participants memorize sequentially presented numbers and indicate if a number is the repetition of the number presented n numbers earlier. A typical test in the animal literature is the delayed match to sample task: An animal has associated two stimuli, for instance, the colors red and blue, with food. At the start of a trial, it sees the color red, which then disappears from view. After a delay, the animal has the choice between a red and a blue food bowl. It chooses the red bowl (using WM) because the condition of the trial (the red color) determines which bowl is baited. The blue bowl, equally associated with food, is not chosen because the information held in WM allows for a goal selection based on the task demands on the current trial. This general protocol has been used in numerous experiments and variations with different species (e.g., Weinstein, 1941; Lu et al., 1993; Zhang et al., 2005; Bloch et al., 2019). The neuroscientific literature interprets physiologically sustained activity during the delay as a correlate of WM, other physiological processes notwithstanding. This concept of “active memory” has shaped our understanding of this cognitive process (Fuster and Alexander, 1971; Funahashi et al., 1989; Goldman-Rakic, 1995; Miller et al., 1996; Fuster, 2000). Sustained activity, of which persistent neuronal spiking is a simple form, differentiates between samples and thereby encodes them as information. The presence or decay of this information during the delay is correlated with correct and incorrect matching of the sample, respectively. The amount of information encoded by neuronal activity is quantifiable, and significant differences between correct and error trials exist that indicate information loss (Brody et al., 2003; Nieder, 2012; Jacob and Nieder, 2014; Veit et al., 2014; Moll and Nieder, 2015). For this article, we will consider the active physiological process of sustained activity during the delay to be the “fingerprint of WM” (Box 1). Its presence can be unequivocally detected by neuronal recordings from animals performing a task. Such sustained activity has been recorded in mammals and birds (e.g., Fuster and Alexander, 1971; Funahashi et al., 1989; Diekamp et al., 2002; Baeg et al., 2003; Veit and Nieder, 2013; Tsutsui et al., 2016; Wu et al., 2020). The similarity of the WM fingerprint in these different species is indicative of a common mechanism (Veit and Nieder, 2013). This definition of WM narrowly focusses on one aspect of WM, it can only explain WM as an effect under equally narrow experimental circumstances (Miller et al., 2018). We chose to focus on this simple physiological definition, with all its limitations, to facilitate comparability between species. The definition is limited to maintenance of information for a short time, and cannot be used to differentiate the many possibilities of how successful behavioral performance emerges in a WM task (Zentall and Smith, 2016), nor can it tease apart the many intricacies of WM function at the physiological level (Miller et al., 2018). But by using an appropriate experimental setup, the physiological measure at the center of our definition is robust and can be controlled for influences on WM (Box 2). This is essential for the comparative approach, to ensure that the measurements in different animal species are always of the raw information-storage, quantifying WM abilities.

How is the definition of WM, and its measurement, informative about general cognitive abilities? Cognition requires a system that processes information to produce behavior. We argue that “the neuron remains the important unit of function for developing a rational account of how behavior is generated” (Barlow, 1995). The mechanisms underlying WM (Miller et al., 2018) are essential for the maintenance and processing of sensory stimuli and the generation of action plans (“executive control”) that are the foundation of flexible behavior. One key aspect of WM is its capacity, as it determines how much information is simultaneously available for processing. By comparing this capacity, we aim to understand, if cognitive abilities are based on the same fundamental resources, or if already on this basic level a divergence occurs that limits some species’ cognitive abilities. Measuring the capacity and complexity of WM could serve as a proxy for measuring the complexity of cognition in general. Indeed, using a battery of different tests, Kolata et al. (2005) quantified the learning and success rate of mice and found that “general cognitive ability” co-varied with spatial WM capacity. In humans too, WM capacity is correlated with fluid intelligence (Cowan et al., 2005; Fukuda et al., 2010; Luck and Vogel, 2013).

## The Role of Contextual Variables in Working Memory Tasks

A precise measurement of the underlying process is required to compare the cognitive abilities of different animal species. This implies that “contextual variables” must not influence the results. A contextual variable is the result of an interaction between species and the test environment. Examples are the motivational state of the animal for an available reward, sensory demands of relevant stimuli, and motoric demands of the behavioral task (Bitterman, 1965; Macphail, 1985). Removing the influence of contextual variables on an animal’s performance is, therefore, essential for comparative research. For example, a food reward used to motivate a pigeon may not motivate a monkey to work in an otherwise identical task, leading to vastly different performances (Macphail, 1985) argued that such contextual variables are the parsimonious explanation for observed performance differences, instead of underlying cognitive differences. Hence, in this example, the appropriate food reward for each animal is required to motivate the same behavioral and cognitive process. Saturation is another variable that determines the motivational drive (Bitterman et al., 1958) found that for their fish, food intake and the number of days of food deprivation are positively correlated, with a few days of deprivation already showing a strong effect on intake, comparable to what has been found for water deprivation in rats (Stellar and Hill, 1952). By using non-differential reward, motivation should only be affected in a general matter (e.g., by saturation). Unfortunately, it’s not always as simple as controlling saturation or switching the food reward. Animals whose ecology is based around active foraging for long periods can be trained comparatively easy because the task design is similar to naturally occurring foraging. Pigeons, corvids, rats, and monkeys are part of this group. Other animals cannot be motivated as readily because testing them in isolation, in what is an ecologically untypical task for them, is prohibitive for any performance in the task. Rewards commonly used in DMTS tasks, small amounts of food or water, might be unsuitable to elicit any kind of motivation (a snake who may only eat once a month and actively hunts for its food might be a striking example for such issues). This means that the reward for matching behavior needs to be adapted to elicit a motivational drive in the tested species, ideally in multiple trials back-to-back. Social or environmental variables that are rewarding to the animal might be an alternative to food-based rewards (e.g., for a snake, escaping a cold place to enter a warm place might be rewarding; for a fish leaving a current and entering still water might be rewarding, etc.). This requires precise knowledge of the animal’s ecological background and creative experimental design to ensure that the animal can only use the sample information to solve the task.

**BOX 3. The “nature” of sustained activity.**

Reward coding signals, motor preparation signals, and retrospective (encoding the memorandum) or prospective (e.g., encoding the identity of the response stimulus, or the response location) content can be encoded by sustained activity. These different interpretations can be dissociated through the experimental design of the DMTS task on both a behavioral level (Zentall and Smith, 2016) and on a physiological level. Motor related signals (i.e., planning on where to respond) can be controlled for by randomizing the response location or limiting it to one kind of response. To avoid that the sustained delay activity reflects differential reward [as it does for monkeys and pigeons (Watanabe, 1996; Browning et al., 2011; Johnston et al., 2017a)], a common-outcome procedure is sufficient, for which sustained neuronal activity then encodes the sample stimulus (Johnston et al., 2017b). If every memorandum of the DMTS task is represented with the same amount of information (i.e., by the same type of representation and all stimuli are equally easy to differentiate from one another), comparing WM capacity between species can be based on this information being encoded in the neuronal representation of WM. Knowing how this information is represented, as sample identity, reward code, or any other form of code, is not required for the measurement of capacity. Decoding neuronal activity during the delay reveals the amount of information in WM (Buschman et al., 2011) and this measure is to be compared between species.

Many tasks that test cognitive abilities were designed for primates and make use of their specific abilities (e.g., manipulations of objects, touchscreens, long periods of training, etc.). Due to contextual variables, the translation of such task designs to the needs of other species can be very challenging. Pigeons, for instance, require many more learning trials than monkeys until they perform equally well on many tasks (Scarf and Colombo, 2020), even though a behavioral protocol might be well established for pigeons. It might, therefore, prove to be virtually impossible to train species like fish on such tasks, entirely because a non-cognitive trait, like a limited trial number due to quick saturation, prevents task acquisition. These tests of impressive higher cognitive abilities are, therefore, often difficult to compare between different species, simply because the tested animal does not produce the cognitive trait at all. The issue might even arise at a more basic level. The lack of a hand to manipulate things, or eyes to see with, will dramatically alter the way an animal cognitively engages with the environment. This raises the question if the WM measures of DMTS tasks, performed with samples of different sensory modalities, can even be compared in a meaningful way. Sensory specialization is commonplace and testing an animal within a sensory dimension it is adapted to is a prerequisite to investigate its WM capacity. This is exemplified by using different modalities in the same species that will yield different performances in DMTS tasks. These differences can be explained by sensory discriminability that is required for differential encoding. A pigeon might not be able to differentiate between two odors, while a rat easily can. However, no meaningful information about the sample can be memorized when the upcoming choice between alternatives does not allow for differentiation. By investigating WM, as code for information about samples, the cognitive process is reduced to the differential translation of sensory input into a neuronal representation. This is a very simple form of cognition that we assume to be present irrespective of sensory-motor adaptation. How much information can be encoded at the same time thus becomes a “pure” capacity for comparison. Such information can even be independent of its sensory origin, exemplified by the neuronal activity of monkeys and crows, where neurons encode the same meaning, rather than just the sensory identity, of stimuli (Wallis et al., 2001; Veit and Nieder, 2013; Moll and Nieder, 2017). By measuring WM abilities, we might be able to quantify differences in cognitive abilities, using a unified testing regime that overcomes hurdles imposed by contextual variables. Such context-free WM abilities allow testing Macphail’s null-hypothesis (Macphail, 1985), supporting it with WM being similar in animals, or disproving it with differences in WM.

## Adequately Measuring WM

The delayed matching to sample (DMTS) task, originally introduced by Blough (1959), has become a benchmark for investigating memory processes (Zentall and Smith, 2016). In DMTS tasks, an animal has to attend to a behaviorally relevant stimulus (“sample”), and following a delay, in which the sample is not present, select a matching stimulus from an array of multiple stimuli. Alternatively, it is also possible that a non-matching stimulus has to be selected (delayed non-match to sample, DNMTS) for an emphasis on control processes (manipulation of information). Both experimental designs require the animal to encode the sample, actively maintain its representation throughout the delay, and choose the matching stimulus (Box 3). Therefore, WM can be measured in the delay period, when the stimulus is not present, isolating the process of active maintenance from sensory processes. This describes the concept of DMTS. The way this task is implemented can differ based on species-specific requirements (see below), making the DMTS suited for many different species. For instance, it can be spatially distributed, with the sample in one location and the match in another, where the delay is defined by the animal covering the distance between sample stimulus and choice array. Or on a touchscreen by attending to the sample and after a delay, matching it in a stimulus-array. The DMTS task alone primarily tests the short term memory of an animal. A component guiding behavior may add the requirement for manipulation of this information in combination with the stimulus, like a rule instructing a response to “same” or “different,” depending on stimulus identity.

We suggest two measures to quantify WM. The capacity of WM (how many items can be stored simultaneously) is indicative of how much available information can be integrated by a cognitive process. This measure can be influenced by chunking individual pieces of information into larger units (Miller, 1956; Cowan, 2001; Cowan et al., 2005). Our approach seeks to quantify WM at the level of differential items to elucidate if differences in capacity exist. Thus, experimental conditions that prohibit chunking are required. A possible outcome of testing raw capacity is to find great similarity between species, as is the case for monkeys and crows (Buschman et al., 2011; Balakhonov and Rose, 2017). Further experiments with conditions that allow chunking may then reveal if all animals can make use of this cognitive strategy. However, successfully testing chunking in non-human animals comes with its own set of challenges (Terrace, 2001; Mathy and Feldman, 2012), especially if a comparative approach is aimed for. Information decay is an indicator of WM performance. This can be measured directly by investigating the neuronal signal, comparing information at the start of the delay (after stimulus offset) to the end of the delay (Buschman et al., 2011). The retention of the sample through a delay needs to be part of the initial training to ensure that the animal learns to maintain the information (Dorrance et al., 2000). Short delays (one to 2 s) facilitate testing of different species because the burden on WM is kept as small as possible while still allowing for meaningful quantification of information. The key to comparability is that sustained activity is present. Short delays that facilitate successful performance, allow for species comparison of the amount of retained information at the end of the delay. Delays can subsequently be prolonged to see how information decays, resulting in task failure. If prolonged retention (e.g., Grant, 1976) is based on sustained activity, a comparison of information at different time points of the delay is possible between animals. If the delay activity is not sustained (but performance is), this would be indicative of other processes not covered by our narrow definition of WM. This can also be tested by adding components to the task that interfere with WM but not with, for example, long term memory (e.g., familiarity Brady and Hampton, 2018). Additional experiments investigating this different process would then be required. By appropriately adapting a DMTS task for different animals, quantification of WM can become a suitable tool to compare cognitive abilities between individuals of one species [as has been shown to work in mice by Kolata et al. (2005)] and ultimately between different species. Measuring WM with a DMTS task is very suitable to neutralize the role contextual variables may play. This is based on three attributes of the test regime: (1) The DMTS task is usable with stimuli of different modalities (visual, olfactory, auditory), thereby eliminating species-specific sensory demands. (2) The task duration can be adapted to match the ecological and ethological time scale of the tested animal, which overcomes hurdles of temporal scaling (e.g., a bird pecking on a touchscreen responds a lot faster than a fish swimming to an answer location). (3) The choice the animal has to make during the matching can be adapted according to the animal’s abilities to indicate a decision (e.g., by touching a choice key, navigating to a choice position, etc.). By adapting the DMTS task to a species specialized adaptations (e.g., using rats’ excellent sense of smell, instead of their comparatively poor vision), tests can make use of the ethological repertoire of the animal instead of forcing it into producing disjointed conditioned responses.

## Working Memory of Different Species: Similarities, Differences, and Comparisons

There is an abundance of studies investigating WM in different species, Lind et al. (2015), list such studies. The primary concern of this article is how the results of the individual studies may support a claim for Macphail’s null-hypothesis in principle. To that end, studies have been selected that fulfill the following criteria. The WM tested in the animal is considered “active” in the sense of this text (i.e., the maintained information is accessible for manipulation while it is not physically available). The experimental design is suitable for adaptation for other animal species (i.e., contextual variables can be neutralized). Alternative explanations of performance can be excluded (e.g., associative memory, stereotypical responses, etc.).

Due to the relative ease of adapting the task design, the WM abilities of monkeys are easiest to compare to humans’. Quantification of WM performance can be assessed with delay length, the number of training trials, and capacity (Weinstein, 1941) showed that rhesus monkeys successfully perform a DMTS, using objects at delay lengths of 5, 10, and 15 s, for a sample size of one. This study simultaneously also tested two young, pre-verbal children in the same task. Both species learned to perform at virtually the same level for all delay lengths, but humans took far fewer trials to learn the procedure. This might reflect a quantitative difference between the species. The number of trials to reach a defined performance threshold in a DMTS task is a good measure for this quantitative difference (Scarf and Colombo, 2020) have suggested the same metric when comparing the performance of monkeys to pigeons. The capacity of WM at stable delay length was investigated by Buschman et al. (2011), who showed in a DNMTS task that macaques perform successfully for short delay lengths of 800–1000 ms with up to five samples. A marked drop in performance occurred at five items, indicating a capacity of about four items, strikingly similar to the famous “magical number four” of human WM (Cowan, 2001; Buschman et al., 2011).

The DMTS and DNMTS protocols are also used in rats and mice. Rats have been shown to successfully learn to discriminate and match stimuli in the visual domain (Mumby et al., 1990; Prusky et al., 2004), but in these experiments, additional factors may interfere with the measurement of WM. Unlike monkeys, rats and mice have poor vision, so visual stimuli are most likely not adequate for testing WM in comparable ways. Additional features of task design (the novelty of stimuli, olfactory cues, and object recognition processes) require special attention to ensure that WM is adequately measured and compared to what is being measured in other species (Ennaceur, 2010). Fortunately, adapting DMTS tasks from the visual to the olfactory domain resolves these issues. When different odors are used as sample and choice stimuli, both rats and mice can perform at very high levels of asymptotic performance and show degrading performance as a function of delay length (Lu et al., 1993; Liu et al., 2014; Roddick et al., 2014).

The range of species is not limited to mammals. Birds were successfully tested in WM paradigms. The physiological correlate of WM has been described in pigeons performing a visual go-no-go task (Diekamp et al., 2002), where pigeons had to maintain an instructive color across a delay and match a behavioral response. Direct comparisons of monkeys and pigeons performing the same task have been performed, using a change detect paradigm (Leising et al., 2013; Wright and Elmore, 2016). The results indicate that there is no major difference between the species. A second bird species adds to these findings. Crows’ WM has been investigated in the visual domain in combination with abstract rules, here too the physiological process of WM has been recorded (Veit and Nieder, 2013; Veit et al., 2014; Balakhonov and Rose, 2017) have explicitly compared WM capacity in crows to monkeys reproducing the task of Buschman et al. (2011) and were able to show that crows and monkeys show the same capacity dependent function of performance, reaching a plateau performance at about four items (Buschman et al., 2011; Balakhonov and Rose, 2017). Overall, the results of WM studies in birds and monkeys indicate virtually identical physiological processes and behavioral performance amongst these two groups of animals (Colombo and Scarf, 2012; Güntürkün, 2012; Güntürkün and Bugnyar, 2016), a result congruent with behavioral observations of cognitive abilities and indicative of convergent evolution (Emery and Clayton, 2004; Jarvis et al., 2005).

Amongst “fish” (a paraphyletic group of animals), few species were tested on their WM abilities. Recently, zebrafish (*Danio rerio*) have been tested in a DMTS task, using different colors with a delay of three and 4 s (Bloch et al., 2019). With their study, Bloch and colleagues established an experimental setup that allows fish to be tested, overcoming contextual hurdles for an animal species that is notoriously difficult to train on a behavioral task. A substantial constraint is the meager amount of trials that can be performed by the fish in a session [in the study of Bloch et al. (2019), only ten trials]. The fish were considered to have learned the task only if they performed at a level of ≥70% correct in a session, for three consecutive sessions, to account for this low number of trials. This study nicely overcomes contextual variables impeding comparative cognition between fish and other animals, by using the DMTS task. Similarly, WM abilities can be quantified even outside of the vertebrate clade. Bees (*Apis mellifera*) can learn to match visual and odor samples after a delay and are able to successfully transfer to novel stimuli (Giurfa et al., 2001; Zhang et al., 2005). Performance of fish and bees is reported to be lower than what can be found in other vertebrates and the length of the delay strongly influences the performance, nonetheless, the principle holds.

The results of mammals, birds, fish, and bees show that WM can be comparably measured across species. Even when they have vastly different organizations of their brains, vastly different ecological niches, and vastly different contextual specialties. Comparisons across species have to be considered carefully nonetheless. WM capacity, retention time, and length of training (measured in trials to criterion) are valuable indicators that allow us to compare the vastly different species sensibly. But these metrics are themselves not completely context-free. The capacity of WM is subject to two competing models of resource allocation, discrete (Luck and Vogel, 1997; Awh et al., 2007) and continuous (Alvarez and Cavanagh, 2004; Bays and Husain, 2008). Depending on which model is being tested in a given experiment, the capacity estimate of WM might vary, based on the applied method of measurement (Fukuda et al., 2010; Luck and Vogel, 2013). Similarly, the quantified amount of training can only be compared if species-specific attributes are taken into account. For example, a singular trial for the pigeon (who performs several hundred per session) is relatively speaking less relevant for performance than a singular trial for a fish (with only ten trials per session). Careful normalization within species may resolve such issues. A final open issue that remains is that WM has not (yet) been shown on the physiological level for all species and thus can only be inferred from the task design if the same “active memory” system is tested in all instances. Ultimately WM can help us resolve the difficulties of application of the famous studies developed for primates, for other vertebrates. With WM and the DMTS task to measure it, we can actively quantify differences in a basic component of all higher cognition and add to the analysis of the principles of animal cognition.

## Conclusion

To understand animal cognition and to investigate Macphail’s null-hypothesis, different approaches can offer insight. Tackling higher cognitive abilities with complex tasks can produce milestones indicative of qualitative differences, while a focus on fundamental aspects of cognition, like WM, with simple tasks, allows us to recognize the quantitative scaling of abilities. The relative simplicity of WM allows us to quantify an animal’s cognitive ability with a unified testing paradigm (the DMTS/DNMTS) that is adaptable to the species of interest, overcoming methodological and contextual hurdles imposed by the complex tasks. Many vastly different species from different classes of vertebrates (mammals, birds, and ray-finned fish) have been successfully tested in the DMTS task, and it is even applicable for an invertebrate like the bee. This is a vital step toward the goal of comparative cognition. A physiological definition, such as active memory, can offer an additional tool beyond behavior to quantify cognition. Importantly, this physiological approach offers precise criteria for comparison along with tools to analyze the underlying processes not only qualitatively but also quantitatively. There is still a lack of physiological evidence of WM in many species (like fish and bees) that unequivocally shows this basic neural process. However, the addition of avian electrophysiology concerning WM has produced results that strongly support the idea that active WM is a universal neural process amongst vertebrates. Concerning Macphail’s null-hypothesis, we conclude that, on the level of WM ability, there does not appear to be a qualitative difference between different vertebrate species. On the quantitative side, differences between species are detectable. The WM of fish and bees seems to be more limited when compared to mammals and birds. This is indicated by the relative difficulty that comes with training them. Data is, however, still lacking and a comprehensive evaluation of WM in different groups of vertebrates, using the same tests and systematically measuring WM capacity, and retention decay of information, along with the physiological correlates of WM, needs to be performed to conclude whether Macpahil’s null-hypothesis can be disproven on this most basic level of cognition.

## Author Contributions

LH and JR conceptualized and wrote the manuscript. Both authors contributed to the article and approved the submitted version.

## Conflict of Interest

The authors declare that the research was conducted in the absence of any commercial or financial relationships that could be construed as a potential conflict of interest.
